# Deep Learning Deformation Initialization for Rapid Groupwise Registration of Inhomogeneous Image Populations

**DOI:** 10.3389/fninf.2019.00034

**Published:** 2019-05-14

**Authors:** Sahar Ahmad, Jingfan Fan, Pei Dong, Xiaohuan Cao, Pew-Thian Yap, Dinggang Shen

**Affiliations:** ^1^Department of Radiology and BRIC, University of North Carolina, Chapel Hill, NC, United States; ^2^School of Automation, Northwestern Polytechnical University, Xi'an, China; ^3^Department of Brain and Cognitive Engineering, Korea University, Seoul, South Korea

**Keywords:** groupwise registration, graph coarsening, deep learning, convolutional neural network, MRI, brain templates

## Abstract

Groupwise image registration tackles biases that can potentially arise from inappropriate template selection. It typically involves simultaneous registration of a cohort of images to a common space that is not specified a priori. Existing groupwise registration methods are computationally complex and are only effective for image populations without large anatomical variations. In this paper, we propose a deep learning framework to rapidly estimate large deformations between images to significantly reduce structural variability. Specifically, we employ a multi-level graph coarsening method to agglomerate similar images into clusters, each represented by an exemplar image. We then use a deep learning framework to predict the initial deformations between images. Warping with the estimated deformations brings the images closer in the image manifold and their alignment can be further refined using conventional groupwise registration algorithms. We evaluated the effectiveness of our method in groupwise registration of MR brain images and compared it against state-of-the-art groupwise registration methods. Experimental results indicate that deformation initialization enables groupwise registration to converge significantly faster with competitive accuracy, therefore facilitates large-scale imaging studies.

## 1. Introduction

Deformable image registration plays a crucial role in applications, such as dose planning in radiation therapy (Castadot et al., 2008; Velec et al., 2011; Gu et al., 2013; Fortin et al., 2014; Cunliffe et al., 2015; König et al., 2016; Samavati et al., 2016; Brock et al., 2017; Flower et al., 2017; Oh and Kim, 2017), motion and deformation modeling of organs (Yang et al., 2008; Cammin and Taguchi, 2010; Schmidt-Richberg et al., 2012; Risser et al., 2013; Li et al., 2016; Meschini et al., 2016), and automatic delineation of the anatomical structures (Gorthi et al., 2011; Arabi and Zaidi, 2017; Wang et al., 2017). In addition, medical practitioners rely on deformable registration for morphometric analysis of anatomical structures (Shi et al., 2009; Matsuda, 2013; Agnello et al., 2016; Joshi et al., 2016), intra-subject structural changes in longitudinal studies (Wu et al., 2012; Csapo et al., 2013; Lee et al., 2017) and analysis of inter-subject anatomical variability (Chen et al., 2017). To date, numerous techniques have been developed for pairwise registration of a moving image and a reference image (Vercauteren et al., 2009; Suh et al., 2011; Hu et al., 2012; Csapo et al., 2013; Razlighi and Kehtarnavaz, 2014; Onofrey et al., 2015; Heinrich et al., 2016; Aganj et al., 2017; Sun et al., 2017; Yang et al., 2017). However, these pairwise registration methods require selecting a particular image as the reference, to which subsequent analyses are biased (Toga and Thompson, 2001).

Groupwise registration methods do not require a pre-specified reference image, but instead automatically determine the hidden common space in an unbiased manner. Groupwise registration techniques typically simultaneously align a cohort of images to a common space (Sabuncu et al., 2009; Spiclin et al., 2012; Wachinger and Navab, 2013). For example, in Joshi et al. (2004) an initial group center is defined by the average of all affine-registered images. The group center is iteratively updated with the average of images registered to it. While this method mitigates bias, it leads to registration inaccuracy as the initial group center is fuzzy. This limitation was addressed in Wu et al. (2011) by constructing a “Sharp-Mean” group center by weighted averaging of the registered images. ABSORB (atlas-building by self organized registration and bundling) (Jia et al., 2010) is a groupwise registration algorithm that warps the images based on their neighboring images. However, ABSORB does not consider the whole image distribution and takes into account only the immediate neighbors of an image. HUGS (hierarchical unbiased graph shrinkage) (Ying et al., 2014) models the image distribution using a graph and formulates groupwise registration as a dynamic graph shrinkage problem where images, represented as nodes, are warped along graph edges. Yet another groupwise registration strategy is by constructing a minimal spanning tree with a root node that gives a minimum overall edge length to all other nodes. The image deformation is estimated by composing all the transformations along the path from a leaf node to the root node (Hamm et al., 2009).

The aforementioned methods assume a single common space and are not designed to deal with heterogeneous populations with large anatomical variations. An inhomogeneous population with large deformations is better represented using multiple group centers and directly warping the images to a single group center is ineffective and inaccurate (Sabuncu et al., 2008; Liao et al., 2012). As a remedy, the population is typically divided into multiple homogeneous subgroups with an atlas constructed for each subgroup for registration (Sabuncu et al., 2009; Ribbens et al., 2010). For example, Wang et al. (2010) cluster the population into subgroups and perform groupwise registration within each subgroup. The center images of the subgroups are then registered using a pyramidal hierarchy. While effective, these methods are computationally expensive and not scalable to large datasets.

In this paper, we present a novel deformation initialization framework to reduce anatomical variations prior to groupwise registration. This removes large structural variations in an inhomogeneous image population so that conventional groupwise registration algorithms can be applied more effectively and accurately. Our initialization framework is formulated as a two-step process: (i) graph coarsening and (ii) deep learning deformation prediction. In the first step, the images are represented using a graph and are clustered via iterative graph coarsening. In the second step, deep learning is employed to estimate the deformations between images in the population according to the hierarchical structure resulting from graph coarsening.

## 2. Methods

Given a diverse dataset of MR brain images with large inter-subject variability, our objectives are to (i) reduce the anatomical variability in a dataset such that the images can be simultaneously registered to a single latent common space and (ii) speed up groupwise registration so that it is scalable to large-scale datasets. To achieve these objectives, we will employ deep learning for predicting large deformations to reduce structural variations so that the images are close enough to be registered efficiently and accurately to a common space.

### 2.1. Deformation Initialization

#### 2.1.1. Multi-Level Graph Coarsening Based Image Clustering

We propose to use multi-level graph coarsening for image clustering. Graph coarsening is used in multi-level graph partitioning to construct smaller graphs by hierarchically combining neighboring vertices (Xiao et al., 2013; Safro et al., 2015). That is, for an *h*-level coarsening, we have |*G*^0^| < |*G*^1^| < … < |*G*^*h*^|, where |*G*^*h*^| denotes the size of the graph at level *h*. Graph coarsening can generally be accomplished by either (i) contraction or (ii) algebraic multigrid (AMG) (Safro et al., 2015) scheme. In the current work, we use AMG graph coarsening (Ruge and Stüben, 1987; Rakai et al., 2012) to split the dataset into image clusters. Let *G*^0^ = (*V*^0^, *E*^0^) be the original graph consisting of a vertex set $V^{0}=\left\{v_{i}|i=1,\ldots ,N_{0}\right\}$, representing images $I=\left\{I_{i}|i=1,\ldots ,N_{0}\right\}$, and an edge set $E^{0}=\left\{e_{ij}|i,j=1,\ldots ,N_{0}\right\}$, representing the similarity between the images. Each edge *e*_*ij*_ is defined for images *I*_*i*_ and *I*_*j*_ and is calculated via normalized cross correlation (NCC) as

(1) $$\begin{array}{l} e_{ij}=\text{NCC}\left(I_{i},I_{j}\right)=\frac{\sum_{x_{0}}\left(I_{i}\left(x_{0}\right)-\bar{I_{i}}\right)\left(I_{j}\left(x_{0}\right)-\bar{I_{j}}\right)}{\sqrt{\sum_{x_{0}}{\left(I_{i}\left(x_{0}\right)-\bar{I_{i}}\right)}^{2}}\sqrt{\sum_{x_{0}}{\left(I_{j}\left(x_{0}\right)-\bar{I_{j}}\right)}^{2}}}, \end{array}$$

where *x*_0_ is a voxel location in the brain region and $\bar{I_{i}}$ and $\bar{I_{j}}$ are mean intensity values of images *I*_*i*_ and *I*_*j*_, respectively. If registration needs to be performed across modalities, information theoretic measures, such as mutual information, can be used. The fine graph *G*^0^ is progressively coarsened with the coarsened graph at level *l* is denoted as $\left(G^{l}=\left(V_{c}^{l},E_{c}^{l}\right)\right)$. The coarsening algorithm is detailed below:

**Step 1:** The connection of vertex *j* with respect to vertex *i* is considered strong if for given ρ ∈ (0, 1]:
  (2) $$\begin{array}{l} |e_{ij}|\geq \rho \times \underset{k\neq i}{\text{max}}|e_{ik}|,\;k=1,\ldots ,N_{l} \end{array}$$
  where *N*_*l*_ is the total number of vertices at level *l*. Note that this criterion is not symmetric with respect to *i* and *j*. With ρ = 1, the connection of the vertex with maximum similarity with vertex *i* is considered strong. This results in a large number of clusters, each with few images. On the other hand, if the value of ρ is too small, then we get too few clusters. We set ρ = 0.95 so that connections with above 95% of the maximum similarity value are considered strong.
**Step 2:** The *desirability* ψ_*i*_ of a vertex to be selected as a coarse vertex is computed as the total number of strong connections with respect to the vertex. The vertex with the largest desirability is designated as a coarse vertex and all vertices that are strongly connected with respect to this coarse vertex are designated as fine vertices.
**Step 3:** The desirability values of vertices strongly connected with respect to fine vertices are increased by 1. The desirability values of vertices strongly connected with respect to coarse vertices are decreased by 1.
**Step 4:** Steps 2 and 3 are repeated until all the vertices are designated as either coarse or fine.
**Step 5:** Steps 1 − 4 are repeated with the coarse vertices with *l* ← *l* + 1 until we get stable graphs (i.e., when the size of the two consecutive graphs is same |*G*^*l*^| = |*G*^*l* − 1^|).

**Figure 1:**
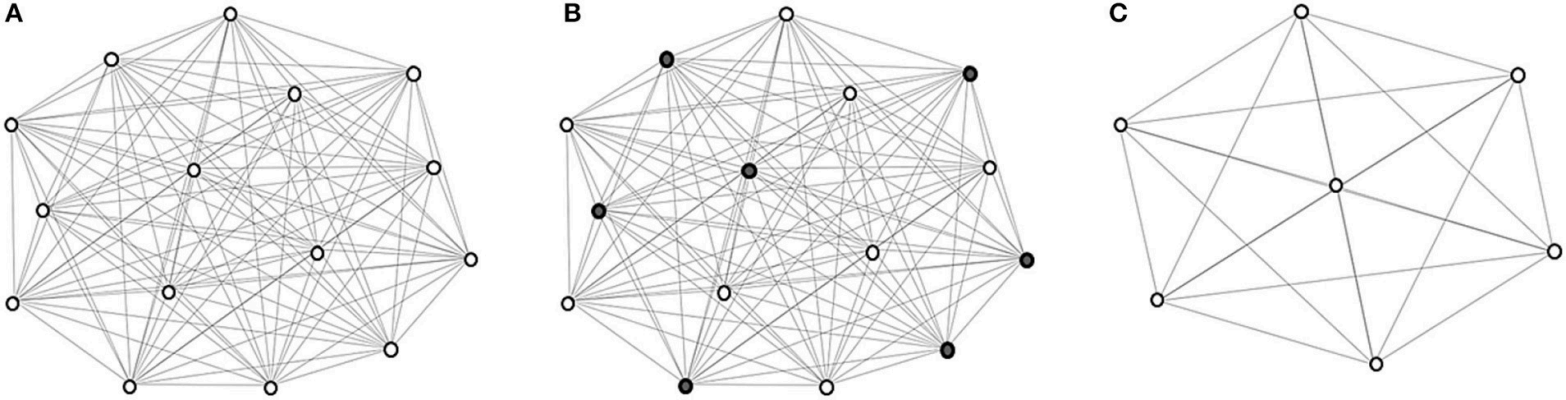
Graph coarsening. **(A)** The initial graph *G*^0^ with vertex set *V*^0^ representing the images in I and edge set *E*^0^ representing the edges between image pairs, computed using (1). **(B)** Coarse vertices (shaded). **(C)** Coarse vertices at a subsequent level.

The coarsening process is illustrated in Figure 1. Eventually, images are hierarchically grouped into clusters, where at each level the cluster exemplars are represented by coarse vertices and cluster members are represented by fine vertices. Cluster exemplars at the highest level will be used for deformation initialization.

#### 2.1.2. Deep Learning Based Registration

**Figure 2:**
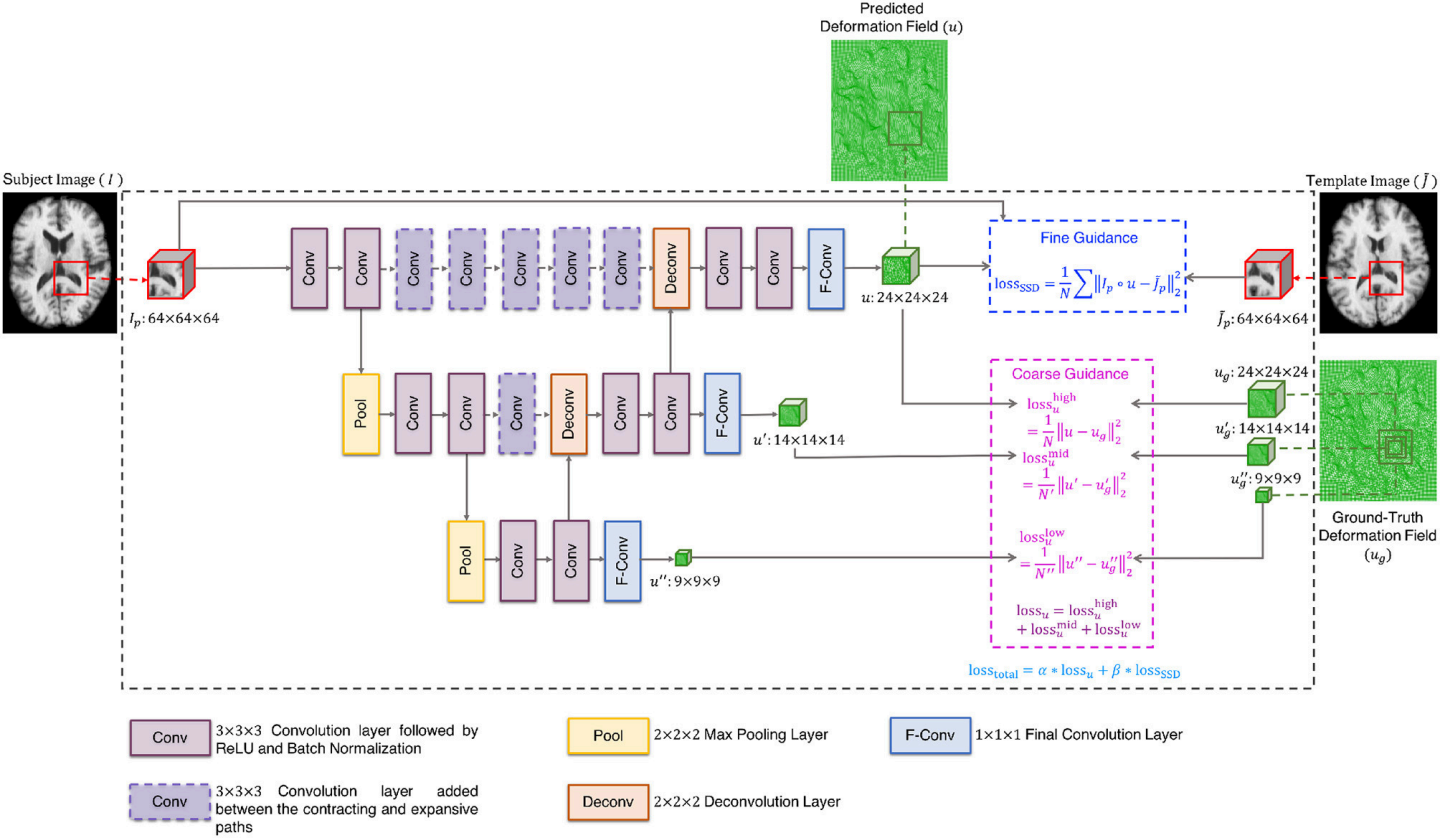
Architecture of registration network.

The cluster exemplars obtained at the highest level (*h*) of graph coarsening, $I^{h}=\left\{J_{j}|j=1,\ldots ,N_{c}^{h}\right\}$, will be used to deform all the images in the dataset. Registration is performed using a convolutional neural network (CNN) (Fan et al., 2018) with these exemplars regarded as fixed templates. The CNN is based on U-Net (Ronneberger et al., 2015), with additional convolutional layers at same levels of contracting and expansive paths to learn high-level features that are helpful in predicting the deformation fields (Figure 2). More specifically, the network consists of (i) 3 × 3 × 3 convolutional layers followed by ReLU and batch normalization, (ii) 2 × 2 × 2 max pooling layers, (iii) 2 × 2 × 2 deconvolutional layers, (iv) 1 × 1 × 1 final convolutional layers, and (v) 3 × 3 × 3 convolutional layers added between the contracting and expansive paths. In addition, a loss function is added in each layer to ensure that the parameters of the frontal convolutional layers are updated. This strategy helps to avoid over-fitting caused by the more frequent parameter update of the later convolutional layers. The registration network takes the overlapping 64 × 64 × 64 patches as input, and outputs 24 × 24 × 24 deformation field patches. In order to obtain a deformation field that is equal in size to the input image, we extract the predicted deformation field patches with a step size of 24 without overlap. A CNN is associated with each template. To train the CNNs, we first select the template which is most similar to all other templates, based on the following criterion:

(3) $$\begin{array}{l} \tilde{J}=\underset{J\in I^{h}}{arg max}\overset{N_{c}^{h}}{\underset{j=1}{\sum }}\text{NCC}\left(J_{j},J\right). \end{array}$$

The CNN is trained using dual-guidance: (i) coarse guidance from deformation fields estimated using an existing registration method and (ii) fine guidance using image dissimilarity between $\tilde{J}$ and the warped subject images. The latter ensures that the training does not completely depend on the guidance from ground-truth deformation fields estimated from the existing registration method. We used diffeomorphic Demons (Vercauteren et al., 2009) to estimate the ground-truth deformation fields. Our learning model is therefore semi-supervised with loss function consisting of two components: (i) the Euclidean distance between the predicted and the ground-truth deformation fields (loss_u_) and (ii) the sum of squared intensity difference between $\tilde{J}$ and the subject image warped using the predicted deformation field (loss_SSD_). As shown in Figure 2, the deformation field is predicted at three different resolution levels, therefore loss_u_ is comprised of the loss functions computed at each level i.e., $\text{los}{\text{s}}_{\text{u}}=\text{los}{\text{s}}_{\text{u}}^{\text{high}}+\text{los}{\text{s}}_{\text{u}}^{\text{mid}}+\text{los}{\text{s}}_{\text{u}}^{\text{low}}$. The two components of the total loss function were dynamically balanced during the training stage (loss_total_ = α ^*^ loss_u_ + β ^*^loss_SSD_). Initially the first component is given a higher weight α to converge quickly and then the prediction is refined by giving more weight β to the second component of the loss function. At each epoch, the sum of the weights of the two components was equal to 1. We trained the network for 10 epochs, which we found enough for convergence.

We used a 75:25 train-test split of the dataset (excluding the templates) and trained the network using the ADAM optimizer with a learning rate of 1 × 10^−2^. Once the network was trained with respect to $\tilde{J}$, the networks for the other templates were trained using transfer learning by initializing the weights with those of the network trained with respect to $\tilde{J}$ and updating the weights using the ADAM optimizer with an overall learning rate of 1 × 10^−7^. We kept a small learning rate as all the CNNs have a common task domain. Transfer learning allows the training of the CNNs to be expedited.

**Figure 3:**
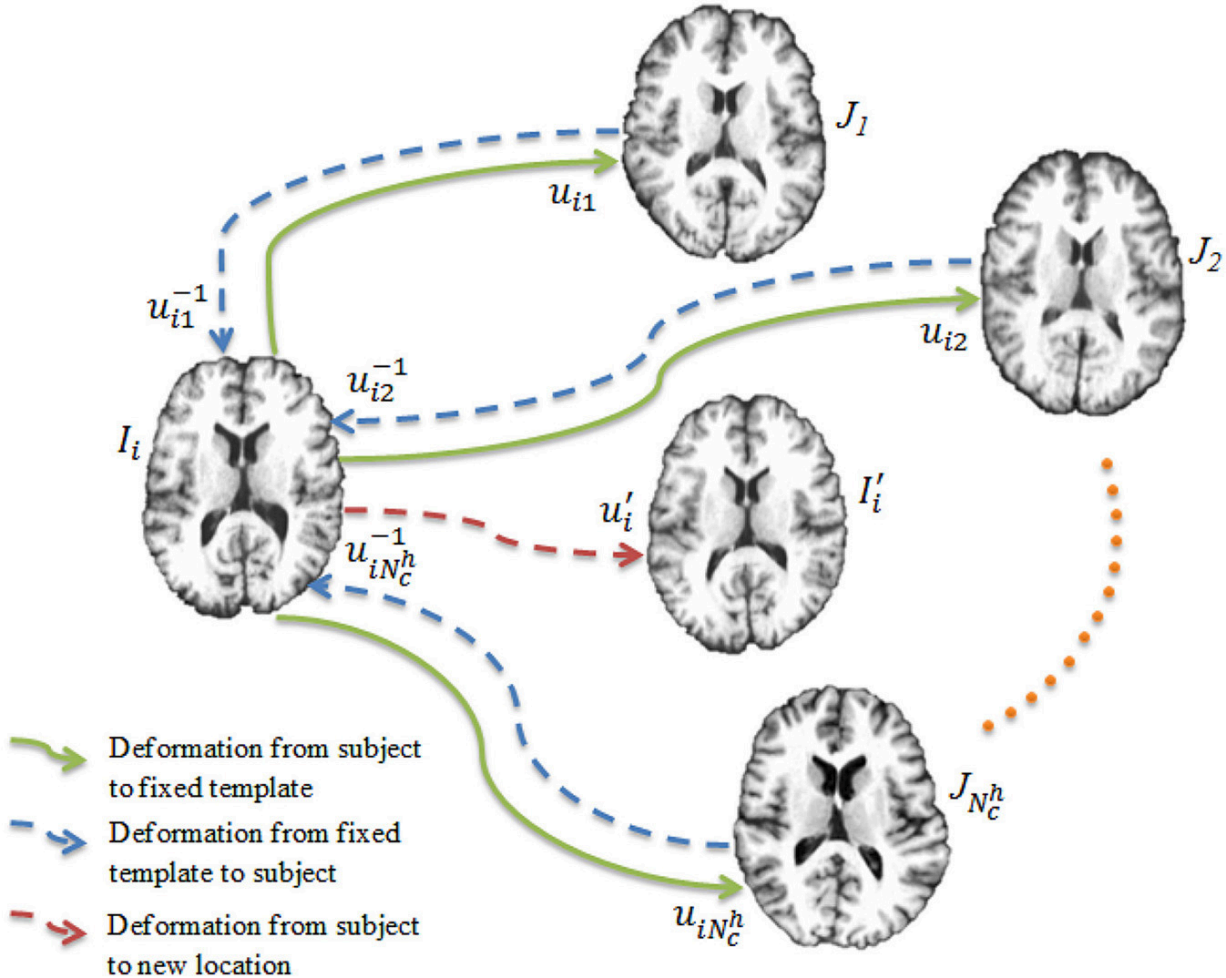
Deep learning registration. Image *I*_*i*_ is registered to $N_{c}^{h}$ templates $\left\{J_{1},\ldots ,J_{N_{c}^{h}}\right\}$ using CNNs. The image $I_{i}^{\prime }$ is warped from *I*_*i*_ using the average deformation field computed using Equation (4).

Once the networks have been trained, each of them is used to register the images to the templates, producing a set of deformation fields $U=\left\{u_{ij}|i=1,\ldots ,N_{0},j=1,\ldots ,N_{c}^{h}\right\}$. Our goal is to warp each image to a hidden common space using the average deformation computed with respect to the templates. To achieve this, we first invert the deformation fields as $U^{-1}=\left\{u_{ij}^{-1}|i=1,\ldots ,N_{0},j=1,\ldots ,N_{c}^{h}\right\}$. The deformation field for an image *I*_*i*_ is computed as

(4) $$\begin{array}{l} {\bar{u}}_{i}={\left(\frac{1}{N_{c}^{h}}\overset{N_{c}^{h}}{\underset{j=1}{\sum }}u_{ij}^{-1}\right)}^{-1} \end{array}$$

and is used to warp the image *I*_*i*_ via $I_{i}^{\prime }=I_{i}\circ {\bar{u}}_{i}$ (see Figure 3). This process is repeated for all the images, producing a set of warped $I\prime =\left\{I_{i}^{\prime }|i=1,\ldots ,N_{0}\right\}$.

The alignment of the images in $I^{\prime }$ can be improved using groupwise registration algorithms. Since the differences between the images are smaller, they can be brought to a common space more efficiently in a smaller amount of time.

## 3. Results and Discussion

### 3.1. Evaluation of Registration Performance

The efficacy of our method was evaluated both qualitatively and quantitatively, in comparison with SharpMean (Wu et al., 2011), ABSORB (Jia et al., 2010), and GroupMean (Joshi et al., 2004). With initialization, the methods are denoted as iSharpMean, iABSORB, and iGroupMean.

**Figure 4:**
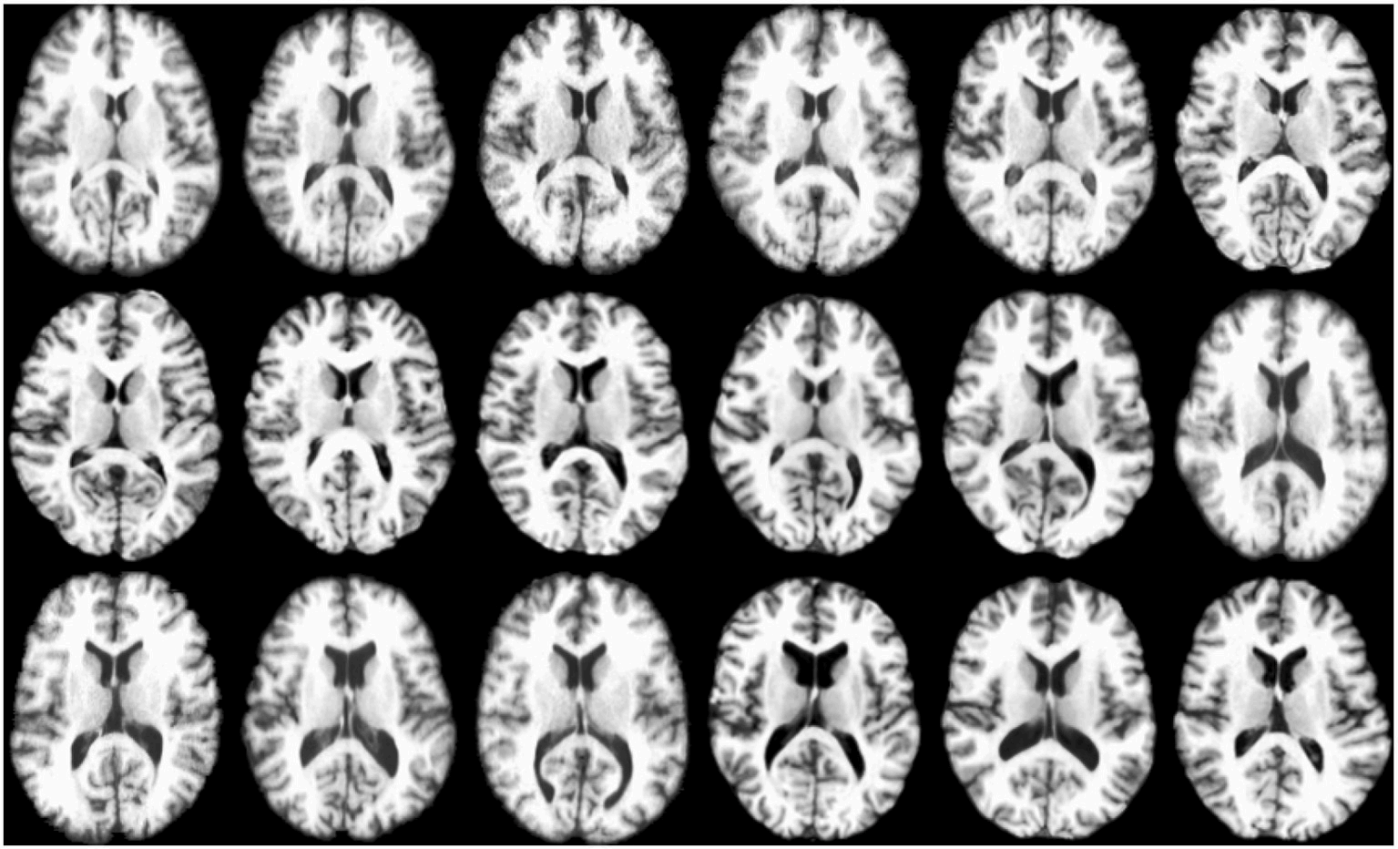
Anatomical variations across images.

The experiments were conducted by combining all the T1 weighted MR images from LONI LPBA40 (Shattuck et al., 2008) and IXI^1^ datasets. As LONI LPBA40 has 40 images and IXI has 30 images, in our dataset we have a total of 70 images that we registered jointly. All the images have 184 slices of 220 × 220 pixels with isotropic voxel size of 1mm^3^. The age range for LPBA40 is 29.20 ± 6.30 years and IXI is 20–54 years. The union of two datasets ensures that the images exhibit large inter-subject variability characterized by the presence of different age groups (young adults and elderly). Figure 4 shows some typical images from this dataset, indicating significant inter-subject differences. All the images were histogram matched and affine registered using ANTs (Avants et al., 2008). The image which is most similar to the rest of the images in the dataset is used as template for affine registration. Also, for training the deep learning based registration network, we had a total of 69 images (excluding the template), which were divided using a 75:25 train-test split. This means that 52 images were used for training and the remaining 17 images were used for testing.

**Figure 5:**
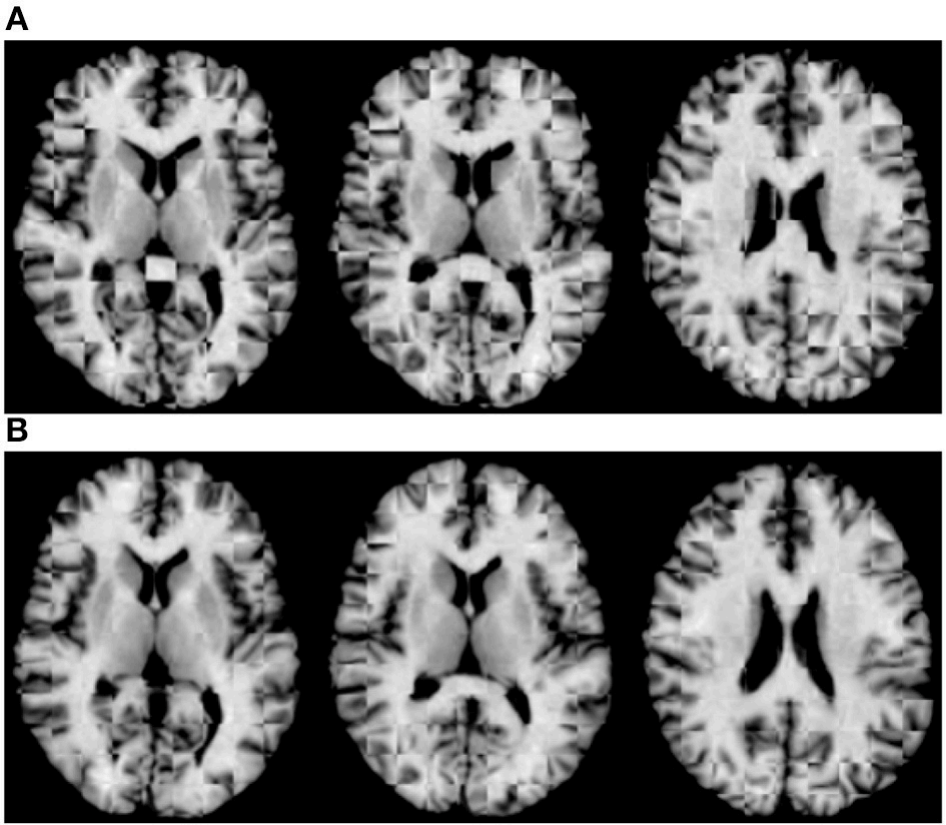
Axial-view checkerboard images **(A)** before and **(B)** after initialization.

For qualitative assessment, we used checkerboard images to simultaneously display two images so that structural boundaries can be compared. In the ideal situation where two images are perfectly aligned, the checkerboard image will be seamless. Figure 5A shows the axial-view checkerboard image of two randomly selected images before initialization, showing apparent misalignments especially in the lateral ventricular region. In contrast, Figure 5B shows that deformation initialization reduces variability across images.

**Figure 6:**
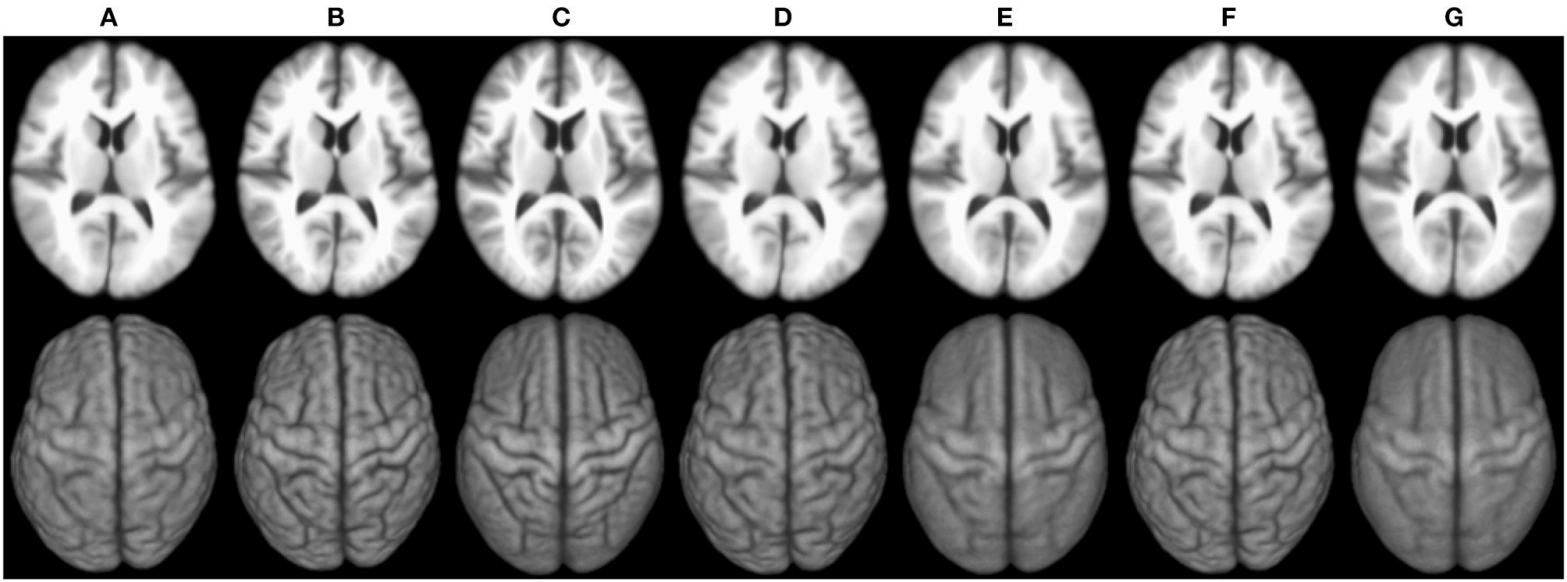
3D surface renderings (*bottom row*) of the group mean images (*top row*) generated by **(A)** deformation initialization, **(B)** iSharpMean, **(C)** SharpMean, **(D)** iABSORB, **(E)** ABSORB, **(F)** iGroupMean, and **(G)** GroupMean.

Figure 6 shows the 3D surface renderings of the group mean images given by different methods. It can be seen that groupwise registration with initialization improves sharpness of the group mean images of SharpMean, ABSORB, and GroupMean.

For quantitative evaluation, we computed the NCCs between all the warped images with respect to the group mean image generated by each method. The results, shown in Table 1, indicate that deformation initialization moves the images closer and groupwise registration with initialization yields higher mean NCC values (statistically significant with paired *t*-tests, *p* < 0.05), than without initialization.

**Table 1:** NCCs (mean ± std) for the various methods.

**Initialization**	**iSharpMean**	**SharpMean**	**iABSORB**	**ABSORB**	**iGroupMean**	**GroupMean**
0.982 ± 0.004	0.997 ± 0.001	0.985 ± 0.004	0.990 ± 0.002	0.983 ± 0.005	0.996 ± 0.001	0.978 ± 0.006

**Figure 7:**
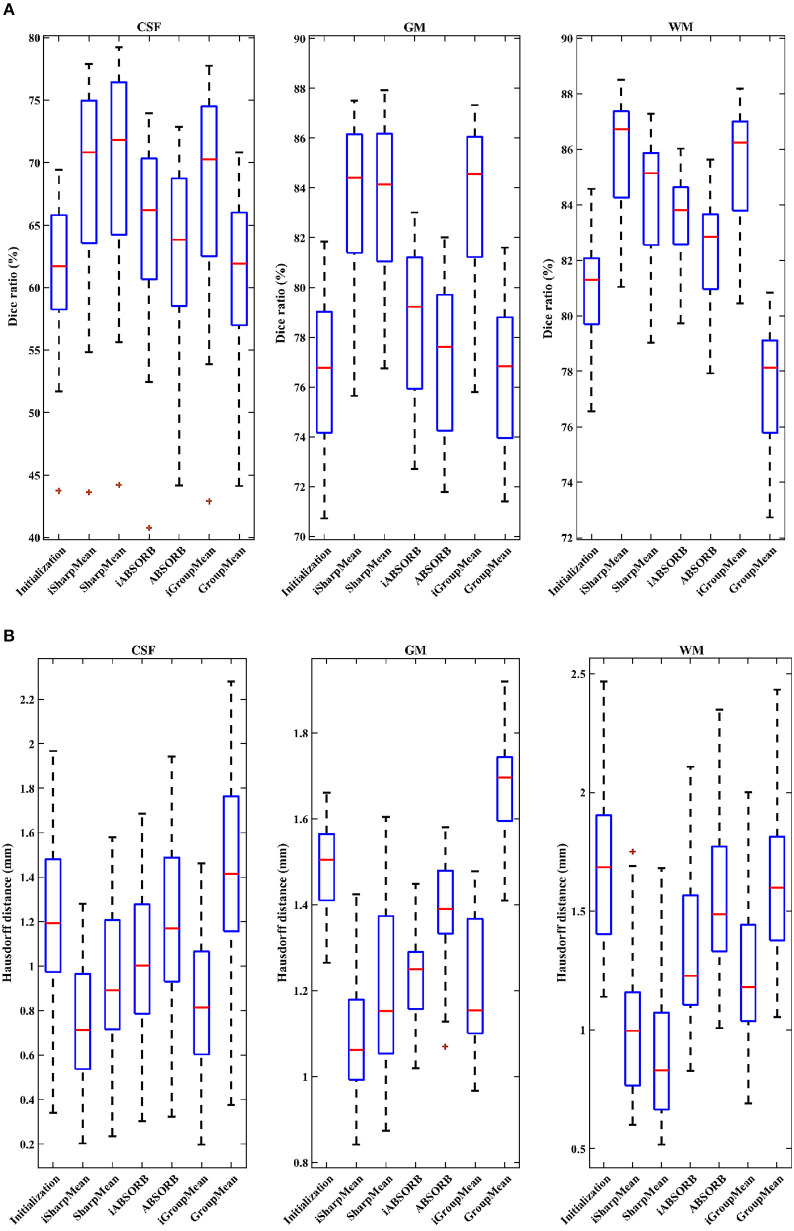
**(A)** Dice ratios (%) and **(B)** 95th percentile of Hausdorff distances for different tissue types (CSF, GM, WM).

Evaluation was also performed based on Dice ratio of hippocampus and brain tissue segmentation, i.e., cerebrospinal fluid (CSF), gray matter (GM), and white matter (WM). The Dice ratio (D) is given by

(5) $$\begin{array}{l} \text{D}=\frac{2|V_{1}\cap V_{2}|}{\left|V_{1}|+|V_{2}\right|}, \end{array}$$

where *V*_1_ is the volume of a segmented tissue or hippocampus in the subject image domain and *V*_2_ is the volume of a segmented tissue or hippocampus in the reference image domain. The reference image for hippocampus and brain tissues is obtained in the common space, respectively by majority voting based on the hippocampus and tissue segmentation of all the warped images. The results for different brain tissues are summarized in Table 2. The overall Dice ratio achieved by deformation initialization is 72.92(±9.16)%. iSharpMean and SharpMean registration methods achieve comparable results with overall values of 79.35(±8.93) and 79.24(±8.15)%, respectively. The differences are not statistically significant (*p* > 0.05). The results for iABSORB and ABSORB are comparable, i.e., 75.66(±8.93) and 74.29(±8.93)%, respectively, and the differences are not statistically significant. iGroupMean [78.93(±9.13)%] yields higher Dice ratios with statistical significance (*p* < 0.05) than GroupMean [71.87(±8.32)%]. The results are summarized using box plots in Figure 7A for CSF, GM, and WM. The Dice ratios for hippocampus are summarized in **Table 4**. The groupwise registration methods with initialization show improved Dice ratio (statistically significant with *p* < 0.05) as compared to no initialization.

**Table 2:** Statistical summary (mean ± std) of Dice ratios (%).

	**Initialization**	**iSharpMean**	**SharpMean**	**iABSORB**	**ABSORB**	**iGroupMean**	**GroupMean**
CSF	61.28 ± 5.30	68.66 ± 7.20	69.86 ± 7.33	64.86 ± 6.36	63.41 ± 6.05	**67.98 ± 7.38**	61.40 ± 5.62
GM	76.55 ± 2.73	83.51 ± 3.06	83.62 ± 3.00	**78.47 ± 2.95**	77.11 ± 2.88	**83.42 ± 3.09**	76.51 ± 2.71
WM	80.92 ± 1.61	**85.87 ± 1.98**	84.23 ± 2.13	**83.65 ± 1.44**	82.34 ± 1.76	**85.39 ± 1.98**	77.68 ± 1.95
Overall	72.92 ± 9.16	79.35 ± 8.93	79.24 ± 8.15	75.66 ± 8.93	74.29 ± 8.93	**78.93 ± 9.13**	71.87 ± 8.32

*Statistically significant improvements (p < 0.05) with or without initialization are marked in bold*.

We computed the 95th percentile of Hausdorff distance for performance evaluation. The Hausdorff distance (HD) is given by

(6) $$\begin{array}{l} \text{HD}\left(R,S\right)=\text{max}\left(\underset{r\in R}{\text{max }}\underset{s\in S}{\text{min}}d\left(r,s\right),\underset{s\in S}{\text{max }}\underset{r\in R}{\text{min}}d\left(r,s\right)\right), \end{array}$$

where *R* and *S* are the 3D point sets of the boundaries of the tissue segmentations or hippocampus of the reference image and subject image, respectively. *d*(*r, s*) is the Euclidean distance between two finite point sets. We reported the 95th percentile of HD since it is less sensitive to outliers. Table 3 summarizes the results for different brain tissue types. The overall value yielded by deformation initialization is 1.37(±0.306)mm thus confirming its usefulness in the reduction of anatomical variability. In addition, deformation initialization improves groupwise registration. Box plots are shown in Figure 7B for evaluation. Table 4 summarizes the 95th percentile of HD for hippocampus. We can see that the initialized groupwise registration significantly decreased the Hausdorff distance as compared to without initialization.

**Table 3:** Statistical summary (mean ± std) of 95th percentile of Hausdorff distances (mm).

	**Initialization**	**iSharpMean**	**SharpMean**	**iABSORB**	**ABSORB**	**iGroupMean**	**GroupMean**
CSF	1.14 ± 0.325	**0.75 ± 0.266**	0.93 ± 0.320	**1.02 ± 0.306**	1.20 ± 0.367	**0.84 ± 0.305**	1.45 ± 0.420
GM	1.42 ± 0.074	**1.09 ± 0.133**	1.19 ± 0.176	**1.22 ± 0.989**	1.40 ± 0.104	**1.22 ± 0.154**	1.68 ± 0.108
WM	1.54 ± 0.289	0.99 ± 0.279	0.90 ± 0.283	**1.33 ± 0.302**	1.55 ± 0.319	**1.24 ± 0.298**	1.62 ± 0.313
Overall	1.37 ± 0.306	0.94 ± 0.276	1.00 ± 0.297	**1.19 ± 0.284**	1.38 ± 0.322	**1.10 ± 0.319**	1.58 ± 0.324

*Statistically significant improvements with or without initialization (p < 0.05) are marked in bold*.

**Table 4:** Quantitative evaluation of hippocampus alignment.

	**Initialization**	**iSharpMean**	**SharpMean**	**iABSORB**	**ABSORB**	**iGroupMean**	**GroupMean**
Dice ratio (%)	71.34 ± 4.37	**72.35 ± 3.98**	71.69 ± 4.71	**71.36 ± 4.43**	70.46 ± 4.93	**71.86 ± 3.98**	69.31 ± 4.66
95th percentile HD (mm)	3.58 ± 0.77	**3.40 ± 0.66**	3.70 ± 0.75	**3.57 ± 0.68**	3.91 ± 0.89	**3.54 ± 0.77**	3.77 ± 0.83

*Statistically significant improvements (p < 0.05) with or without initialization are marked in bold*.

Table 5 summarizes the computational times of all the methods along with the number of iterations needed for convergence. Groupwise registration methods with initialization are faster and converge very quickly, compared with no initialization. More specifically, SharpMean took around 18 h, whereas iSharpMean converged within 3 h with comparable accuracy. iABSORB achieved results comparable to ABSORB and requires 21 h less. iGroupMean took just half an hour to converge, compared with 13.5 h taken by GroupMean. These results indicate that initialization improves registration accuracy by reducing anatomical variability and is hence important for detection of subtle changes associated with aging and disorders.

**Table 5:** Computational times (h) and iteration counts.

	**Initialization**	**iSharpMean**	**SharpMean**	**iABSORB**	**ABSORB**	**iGroupMean**	**GroupMean**
Time (h)	1	3	18	2	23	0.5	13.5
Iteration (#)	1	3	10	1	9	1	15

### 3.2. Significance of Graph Coarsening

To investigate the impact of graph coarsening on template selection, we performed two experiments.

In the first experiment, instead of utilizing graph coarsening, we used randomly selected templates for deformation initialization. The number of selected templates was kept consistent with that given by graph coarsening. It can be observed from Table 6 that the accuracy decreases in comparison with initialization using graph coarsening. This demonstrates the importance of taking into consideration the image distribution in template selection.

**Table 6:** Statistical summary (mean ± std) of Dice ratios (%) for different tissue types.

	**CSF**	**GM**	**WM**	**Overall**
Graph coarsening	61.28 ± 5.30	76.55 ± 2.73	80.92 ± 1.61	72.92 ± 9.16
Random selection	55.07 ± 4.64	73.75 ± 2.79	78.05 ± 2.15	68.91 ± 10.54
Single template ($\tilde{J}$)	61.51 ± 5.57	**76.73 ± 3.10**	**82.18 ± 1.92**	**73.47 ± 9.57**

*Statistically significant improvements (p < 0.05) as compared to graph coarsening are marked in bold*.

In the second experiment, we evaluated the effects of the number of templates. Using a single template (i.e., $\tilde{J}$), although giving good alignment (Table 6), will affect subsequent population analysis [e.g., voxel-based morphometry (VBM)] with bias toward the selected template and neglecting inter-subject variation. Moreover, if the selected template image is an outlier, population analysis can be severely affected. Graph coarsening takes into account inter-subject heterogeneity and determines multiple images that are representative of image sub-populations. The higher Dice ratios given by single template case is partially due to the greater image sharpness when no averaging is performed.

### 3.3. Generalizability

**Figure 8:**
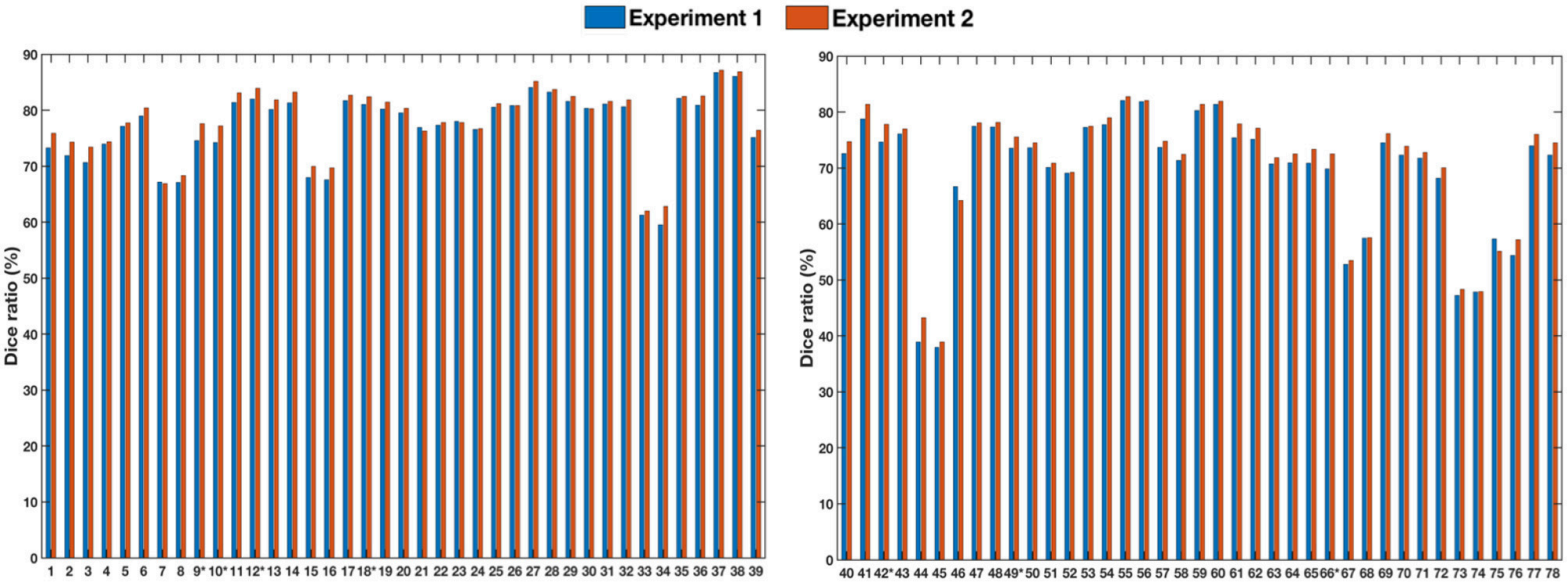
Dice ratios for different ROIs of IXI dataset. “^*^” indicates statistically significant improvements (*p* < 0.05).

To assess generalizability, we conducted two experiments. In the first experiment, we trained the registration network with the LONI LPBA40 dataset and tested it with the IXI dataset. In the second experiment, we trained the registration network with both LONI LPBA40 and IXI datasets and tested it on the IXI dataset. Figure 8 shows the Dice ratios for 78 ROIs (see Table 7) of the IXI dataset. The overall Dice ratio achieved in first and second experiment is 73.07(±9.91) and 74.29(±9.84)%, respectively (*p* > 0.05), indicating generalizability of our method to the unseen image datasets.

**Table 7:** List of 78 ROIs of the IXI dataset.

**ID**	**ROI**	**ID**	**ROI**	**ID**	**ROI**
1	R Hippocampus	27	L Posterior temporal lobe	53	L Inferior frontal gyrus
2	L Hippocampus	28	R Posterior temporal lobe	54	R Inferior frontal gyrus
3	R Amygdala	29	L Remainder of parietal lobe	55	L Superior frontal gyrus
4	L Amygdala	30	R Remainder of parietal lobe	56	R Superior frontal gyrus
5	R Medial anterior temporal lobe	31	L Caudate nucleus	57	L Post-central gyrus
6	L Medial anterior temporal lobe	32	R Caudate nucleus	58	R Post-central gyrus
7	R Lateral anterior temporal lobe	33	L Nucleus accumbens	59	L Superior parietal gyrus
8	L Lateral anterior temporal lobe	34	R Nucleus accumbens	60	R Superior parietal gyrus
9	R Gyri Hippocampalis et ambiens	35	L Putamen	61	L Lingual gyrus
10	L Gyri Hippocampalis et ambiens	36	R Putamen	62	R Lingual gyrus
11	R Central superior temporal gyrus	37	L Thalamus	63	L Cuneus
12	L Central superior temporal gyrus	38	R Thalamus	64	R Cuneus
13	R Medial and inferior temporal gyri	39	L Pallidum	65	L Medial orbital gyrus
14	L Medial and inferior temporal gyri	40	R Pallidum	66	R Medial orbital gyrus
15	R Lateral occipitotemporal gyrus	41	Corpus callosum	67	L Lateral orbital gyrus
16	L Lateral occipitotemporal gyrus	42	R Lateral ventricle, frontal horn	68	R Lateral orbital gyrus
17	L Insula	43	L Lateral ventricle, frontal horn	69	L Posterior orbital gyrus
18	R Insula	44	R Lateral ventricle, temporal horn	70	R Posterior orbital gyrus
19	L Lateral remainder of occipital lobe	45	L Lateral ventricle, temporal horn	71	L Subgenual anterior cingulate gyrus
20	R Lateral remainder of occipital lobe	46	Third ventricle	72	R Subgenual anterior cingulate gyrus
21	L Anterior cingulate gyrus	47	L Precentral gyrus	73	L Subcallosal area
22	R Anterior cingulate gyrus	48	R Precentral gyrus	74	R Subcallosal area
23	L Posterior cingulate gyrus	49	L Straight gyrus	75	L Pre-subgenual anterior cingulate gyrus
24	R Posterior cingulate gyrus	50	R Straight gyrus	76	R Pre-subgenual anterior cingulate gyrus
25	L Middle frontal gyrus	51	L Anterior orbital gyrus	77	L Anterior superior temporal gyrus
26	R Middle frontal gyrus	52	R Anterior orbital gyrus	78	R Anterior superior temporal gyrus

## 4. Conclusion

In this paper, we presented an effective and efficient deformation initialization method for groupwise registration of images with large anatomical differences. Deformation initialization decreases structural discrepancies and brings the images closer to the common space. The results validated that deformation initialization improves alignment accuracy and significantly reduces computation times.

## Author Contributions

SA implemented the code, performed experiments, and prepared the manuscript draft. JF contributed in code implementation. PD and XC helped in designing the experiments. JF, PD, and XC participated in idea discussion. P-TY contributed in algorithm development and critical revision of the manuscript. DS designed the project, led the team, and revised the manuscript.

### Conflict of Interest Statement

The authors declare that the research was conducted in the absence of any commercial or financial relationships that could be construed as a potential conflict of interest.

## Footnotes

^1^Available online at http://brain-development.org/ixi-dataset/
